# Prolonged duration of action of rocuronium in a preterm infant: a case report

**DOI:** 10.3389/fped.2026.1776762

**Published:** 2026-04-07

**Authors:** T. Willegers, K. de Graaff-Korf

**Affiliations:** Department of Neonatal Intensive Care, Isala Clinics, Zwolle, Netherlands

**Keywords:** neonates, neuromuscular monitoring, preterm neonates, prolonged neuromuscular blockade, rocuronium

## Abstract

Prolonged duration of action of rocuronium is rare but has been more frequently reported in infants. Yet it has not been reported in pre-term infants. We present a case of unusually prolonged neuromuscular blockade following a bolus dose of rocuronium administered during rapid sequence induction in a preterm infant leading to difficult weaning from mechanical ventilation. This case highlights the importance of neuromuscular blockade monitoring even after a bolus dose of neuromuscular blockade in (pre-term) neonates.

## 1 Introduction

Neuromuscular blocking agents (NMBAs) are commonly used to facilitate intubation in both adult and pediatric patients in the operating room (OR) or intensive care unit (ICU). However, their use in the Neonatal Intensive Care Unit (NICU) setting is less frequent. Rocuronium, a commonly employed NMBA, exhibits a dose-dependent duration of action when used as a bolus prior to induction. It is well established that the onset time of rocuronium is shorter in neonates compared to children and adults, whereas the mean duration of action is prolonged in neonates (1). This phenomenon is explained by the immature and structurally distinct neuromuscular junction in neonates, differences in postjunctional acetylcholine receptor subtypes, and the prolonged opening time of fetal receptors resulting in enhanced depolarizing potentials (2–8). We report a case of an unusually prolonged neuromuscular blockade following emergency induction with rocuronium in a preterm infant, which contributed to difficulties in weaning from mechanical ventilation.

## 2 Manuscript

### 2.1 Case presentation

The patient was a female preterm infant born spontaneously at 34 weeks and 6 days of gestation as the second twin of a dichorionic diamniotic pregnancy with a birth weight of 2.86 kg (P86) at a local hospital. The Apgar scores were 3,7,8 at 1,5 and 10 min postpartum, respectively. Umbilical cord bloodgas showed a pH of 7.30, bicarbonate 24 mmol/L, base excess −2.7 and a lactate 2.2 mmol/L. The infant experienced initial respiratory distress, necessitating five insufflation ventilations, followed by assisted ventilations. At four minutes post-delivery, oxygen saturations were around 80%. Saturation improved quickly to 98% after assisted ventilation with 100% oxygen. The infant was placed on the mother's chest. Within two minutes, the infant desaturated and required ventilatory support.

Upon transfer to the pediatric ward, the infant desaturated again and was suctioned with removal of copious bloody secretions. Breath sounds were diminished, and ventilation continued with 100% oxygen following oropharyngeal airway placement. Despite high inspiratory pressures (∼30 cmH2O) and PEEP of 10 cmH2O, oxygen saturation declined to 60%. Chest radiography excluded pneumothorax and showed no other abnormalities. The anesthesiologist performed rapid sequence intubation using etomidate (0.3 mg) and rocuronium (3 mg, corrected for birth weight 1.08 mg/kg).

The infant was stabilized and transferred to our NICU. Due to the administration of short-acting sedatives and NMBAs, morphine was initiated before transport as bolus of 0.3 mg intravenous. No continuous morphine for comfort was started after the bolus. Upon NICU admission, ventilation was provided in synchronized intermittent positive pressure ventilation (SIPPV) mode with inspiratory pressures of 21 cmH2O and PEEP of 8 cmH2O without supplemental oxygen. Over subsequent hours, pressures were reduced to 14 cmH2O inspiratory and 6 cmH2O PEEP. Approximately 10 h postpartum, endotracheal continuous positive airway pressure (CPAP) was attempted as spontaneous triggering was observed; however, the infant rapidly became apneic and cyanotic, necessitating return to SIPPV. Over the following hours, intermittent ventilator triggering was observed at the lowest trigger sensitivity, with reduced spontaneous movements compared to other neonates.

Initial respiratory difficulties at birth were attributed to poor transitional adaptation, which resolved rapidly after intubation. In evaluation of the apnea and delayed weaning from mechanical ventilation, cranial ultrasound showed no intracranial pathology, and there was no evidence of perinatal asphyxia. Empiric antibiotic therapy with penicillin and gentamicin was initiated, as infection could not be fully excluded; however, there were no maternal risk factors or clinical signs of infection. Respiratory pathology seemed unlikely given the steadily decreasing ventilatory pressures, absence of oxygen requirement, normal blood gas values, and symmetric breath sounds on auscultation. Laboratory results showed no metabolic or electrolyte disturbances, and no maternal medications were identified that could account for the clinical presentation.

After excluding alternative causes of apnea, partial neuromuscular blockade due to high NMBA dosing and potential prolonged metabolism related to hepatic immaturity was considered. Sugammadex was administered 17 h postpartum at a dose of 2 mg/kg (5.6 mg), resulting in improved spontaneous movements and ventilator triggering. After 20 min a second 2 mg/kg dose further enhanced triggering, allowing a subsequent successful trial of endotracheal CPAP without apnea. The infant was extubated and required no further respiratory support, being discharged the following day.

### 2.2 Discussion

Prolonged duration of action of rocuronium in neonates is uncommon, with few reports documenting this phenomenon, and none specifically in preterm infants (9–12). A recent systematic review by Vanlinthout demonstrated that the duration of action of nondepolarizing neuromuscular blocking agents in neonates is markedly prolonged and highly variable compared to older ages (13). It highlights the importance of neuromuscular blockade monitoring in this population.

In surgical settings, train-of-four (TOF) stimulation is employed to monitor neuromuscular blockade depth and guide sedation discontinuation. However, quantitative TOF monitoring remains limited in infants and children, primarily due to equipment availability constraints (14). Pediatric anesthesia literature and guidelines offer scarce recommendations regarding optimal neuromuscular monitoring and the incidence of residual blockade in this population (15).

Soffer et al. demonstrated the feasibility of TOF nerve stimulation in infants, reporting prolonged recovery of neuromuscular blockade in NICU patients, with median recovery time of 14 h to TOF ratios >70% (12). In our NICU, NMBA use and neuromuscular monitoring are infrequent.

In our NICU, neuromuscular function monitoring was not available, and consequently, quantitative neuromuscular assessment could not be performed. As a result, it cannot be stated with certainty that residual neuromuscular blockade was responsible for the difficult weaning observed. Nevertheless, an immediate marked clinical improvement was noted following the administration of sugammadex, after which the process of weaning from mechanical ventilation proceeded rapidly and uneventfully. This observation supports the conclusion that a degree of residual neuromuscular blockade was likely still present. Measurement of plasma rocuronium concentrations was considered as part of the diagnostic evaluation; however, this was not pursued owing to the anticipated delay in obtaining results and the potential adverse consequences, including prolonged mechanical ventilation and an extended stay in the intensive care unit.

### 2.3 Conclusion

Clinicians should be aware that neuromuscular blocking agents in neonates and preterm infants may exhibit a longer and more variable duration of action than the often-cited duration of approximately one hour (1). This case illustrates that prolonged rocuronium-induced neuromuscular blockade contributed to prolonged mechanical ventilation dependency in our patient, underscoring the critical importance of neuromuscular blockade monitoring following NMBA administration in this vulnerable population, given the potential for extended recovery times.

## Data Availability

The original contributions presented in the study are included in the article/supplementary material, further inquiries can be directed to the corresponding author.
